# The activity of superoxide dismutases (SODs) at the early stages of wheat deetiolation

**DOI:** 10.1371/journal.pone.0194678

**Published:** 2018-03-20

**Authors:** Gracjana Leonowicz, Kamil F. Trzebuniak, Paulina Zimak-Piekarczyk, Ireneusz Ślesak, Beata Mysliwa-Kurdziel

**Affiliations:** 1 Department of Plant Physiology and Biochemistry, Faculty of Biochemistry, Biophysics and Biotechnology, Jagiellonian University in Krakow, Krakow, Poland; 2 Department of Stress Biology, The Franciszek Górski Institute of Plant Physiology, Polish Academy of Sciences, Krakow, Poland; University of Hyderabad School of Life Sciences, INDIA

## Abstract

Unbound tetrapyrroles, *i*.*e*. protochlorophyllide (Pchlide), chlorophyllide and chlorophylls, bring the risk of reactive oxygen species (ROS) being generated in the initial stages of angiosperm deetiolation due to inefficient usage of the excitation energy for photosynthetic photochemistry. We analyzed the activity of superoxide dismutases (SODs) in etiolated wheat (*Triticum aestivum*) leaves and at the beginning of their deetiolation. Mn-SOD and three isoforms of Cu/Zn-SODs were identified both in etiolated and greening leaves of *T*. *aestivum*. Two Cu/Zn-SODs, denoted as II and III, were found in plastids. The activity of plastidic Cu/Zn-SOD isoforms as well as that of Mn-SOD correlated with cell aging along a monocot leaf, being the highest at leaf tips. Moreover, a high Pchlide content at leaf tips was observed. No correlation between SOD activity and the accumulation of photoactive Pchlide, *i*.*e*. Pchlide bound into ternary Pchlide:Pchlide oxidoreductase:NADPH complexes was found. Cu/Zn-SOD I showed the highest activity at the leaf base. A flash of light induced photoreduction of the photoactive Pchlide to chlorophyllide as well as an increase in all the SODs activity which occurred in a minute time-scale. In the case of seedlings that were deetiolated under continuous light of moderate intensity (100 μmol photons m^-2^ s^-1^), only some fluctuations in plastidic Cu/Zn-SODs and Mn-SOD within the first four hours of greening were noticed. The activity of SODs is discussed with respect to the assembly of tetrapyrroles within pigment-protein complexes, monitored by fluorescence spectroscopy at 77 K.

## Introduction

It is commonly known that plants absorb light to drive photosynthesis. However, angiosperm plants also require light for the biosynthesis of chlorophyll (Chl), the main photosynthetic pigment as reviewed in [1–3]. Due to evolution, they have retained only a light-dependent protochlorophyllide oxidoreductase (EC 1.3.1.33; LPOR) to catalyze the reduction of protochlorophyllide (Pchlide) to chlorophyllide (Chlide) [4,5]. This photoenzyme is not activated in the absence of light, which stops Chl biosynthesis and strongly influences seedling development. Seedlings that germinate and grow in darkness follow the developmental pattern known as skotomorphogenesis, as reviewed by Solymosi and Schoefs [2], and become etiolated. On the cellular level, they contain etioplasts with the characteristic membrane structure, prolamellar bodies (PLBs), but no chloroplasts. Even though PLBs contain some proteins of photosynthetic complexes [6–8], these complexes are not assembled and no photosynthetic activity can be observed. Pchlide accumulates in a controlled way, as reviewed in [3,9] and to some extent it is bound within photoactive substrate-enzyme complexes, *ie*. Pchlide:LPOR:NADPH ternary complexes, termed photoactive Pchlide, as reviewed in [5,10–12], where it can be reduced to Chlide even with a single flash of light of a millisecond’s duration. Pchlide molecules, which stay unbound to LPOR, are not converted to Chlide with a short flash and are termed nonphotoactive Pchlide. However, they may serve as the substrate for photoreduction upon continuous illumination.

Light-induced Pchlide reduction triggers the deetiolation (greening) process that leads finally to the formation and the assembly of the functioning photosynthetic complexes, which is accompanied by etioplast to chloroplast transformation and altogether switches the plant to an autotrophic lifestyle [9,13]. Deetiolation is a complex process comprising many well-orchestrated biochemical processes: gene transcription, the biosynthesis of pigments, lipids and proteins *etc*. [14]. The initial steps of deetiolation, which occur in a minute time-scale, include the release of Chlide from enzyme-product complexes, Chl formation, regeneration of the photoactive Pchlide:LPOR:NADPH complexes and dispersion of PLBs [15–23]. Next, the formation of lamellar membranes followed by the formation of stacked thylakoids, the massive synthesis of proteins and Chl, the development of photosynthetic complexes and their activity is observed within an hour time-scale. The time-sequence and the extent of these processes depend on the plant species and on the environmental conditions [24–28].

Etiolation and deetiolation processes as well as the photosynthetic activity are light-regulated via plant photoreceptors phytochromes and cryptochromes [13,29–31, and references therein]. In particular, expression of many enzymes involved in Chl biosynthesis, including LPOR, undergo regulation via phytochrome signalling route, mainly phytochrome A [32–35].

Chls and other tetrapyrroles, which possess conjugated double bonds and hence efficiently absorb light of characteristic wavelengths, can act as photosensitizers [36–38]. Thus, photooxidative stress is a constant companion of a photosynthetic lifestyle [36,39,40]. Reactive oxygen species (ROS), such as superoxide anion radical (O_2_^•-^), singlet oxygen (^1^O_2_) and hydrogen peroxide (H_2_O_2_), produced mainly in chloroplasts [41], are harmful for the photosynthetic apparatus. On the other hand, there is an increasing evidence that ROS are central players in the plant cell signalling network [40,42–46]. It should also be underlined that tetrapyrroles are regarded as contributors to ROS signalling [36]. Photosynthetic organisms have developed various ROS scavenging mechanisms to counteract their toxicity and maintain a balance between their production and scavenging. Some antioxidants like ascorbate, glutathione, α-tocopherol and carotenoids are involved in non-enzymatic protection [39,47,48]. At the same time, the ROS-scavenging enzymes superoxide dismutase (SOD), catalase (CAT) and peroxidases (POXs) are also involved [39,42,47,48].

SOD (EC 1.15.1.1) is a metalloenzyme and catalyzes the dismutation of O_2_^•-^ to less reactive products *i*.*e*. O_2_ and H_2_O_2_:

$$2H^{+}+2O_{2}{}^{•‑}\to H_{2}O_{2}+O_{2}$$

SOD constitutes the front-line of defence against ROS and oxidative stress in plant cells [47,49]. Changes in SOD encoding gene transcript level and SOD activity [50–55] are regarded as indicators of the level of ROS production and oxidative stress [39,42]. Various stress factors, such as salinity, drought, pathogens, high light and many others were observed to increase the activity of SOD and other antioxidant enzymes in numerous studies, *e*.*g*. [50,56]. It was also observed that the induction of antioxidant enzymes, including SOD, is important for the development of plant stress tolerance [50,56–58]. Depending on the metal bound in the catalytic active site, three SOD classes in plants were identified: manganese SOD (Mn-SOD), copper/zinc SOD (Cu/Zn-SOD) and iron SOD (Fe-SOD) [49,59]. Cu/Zn-SOD is the most plentiful SOD form in plant cells, detected in numerous cell compartments: chloroplasts [60–62], mitochondria [63,64], cytosol [54,63,65–67], peroxisomes [63,68,69] as well as in the apoplast [65,67,70]. Fe-SOD is mainly regarded as a chloroplastic enzyme [61,62,71,72]. Mn-SOD was already localized in mitochondria [63,64,73,74] and in peroxisomes [63,75–77].

Maintaining a balance between ROS production and detoxification is particularly important during the deetiolation process. The accumulation of Pchlide, which is a porphyrin, in etiolated seedlings and the rapid synthesis of Chlide and Chl, which are chlorins, bring the risk of uncontrolled excitation of these tetrapyrroles, leading to photooxidation and the production of reactive oxygen species (ROS) [78]. Singlet oxygen production upon the illumination of etiolated *Arabidopsis thaliana flu* mutant, which overaccumulates nonphotoactive Pchlide, has been described previously [79]. ROS-provoked bleaching was also observed in etiolated pea epicotyls [80,81] and in the innermost leaves of cabbage [82], and it was shown that nonphotoactive Pchlide monomers accumulated in these tissues acted as sensibilisers of these reactions. On the other hand, the aggregated ternary Pchlide:POR:NADPH complexes and the paracrystalline PLB structure increase the extent of Pchlide photoreduction and decrease the photosensitivity of the pigments [10], although the PLB structure is dispersed within up to half an hour of the onset of illumination. Attention has also been drawn to the photoprotective role of LPOR [83–85]. ELIP proteins, which are transiently induced in the first hours of deetiolation [86–88] and share a domain with light-harvesting proteins (LHC) [89,90], were suggested to be involved in temporary Chl binding during early deetiolation [89]. They were proposed to play a role in photoprotection during the greening process [91,92], although this was later not confirmed [93]. Recently, it has been demonstrated that ELIPs function as a sensor of Chl overproduction and prevent photooxidation this way [94]. However, it has not been elucidated yet how plants mitigate ROS production at the early stages of deetiolation, at which large amounts of Chl *a* and Chl *b* are synthesized, whereas photosynthetic reaction centers and antenna complexes, as well as the thylakoid structure and photosynthetic electron transport chain, are not yet fully arranged. Altogether, this brings the risk of excitation energy for photosynthetic photochemistry being used inefficiently.

Taking into account that SODs act as the front line of defence against ROS and oxidative stress in plant cells, in our study we focussed on the activity of SOD forms in etiolated wheat (*Triticum aestivum*) leaves, and as a response to the flash-induced deetiolation or deetiolation carried out in continuous light. The first triggers only the photoreduction of photoactive Pchlide, whereas the latter provided a complete photoreduction of the accumulated Pchlide. Wheat leaves are frequently used as a model for studying the deetiolation process because they accumulate plastids that show a developmental gradient from the pregranal plastids at the leaf base to the senescing etioplasts at the leaf tip [22,95,96]. We were interested in verifying whether the onset of deetiolation triggers ROS defence mechanisms and whether the response depends on the developmental stage of plastids. The next query was whether the accumulation of Pchlide in the form of photoactive Pchilde:LPOR: NADPH complexes influences the SODs activity.

## Materials and methods

### Chemicals

All the chemicals were purchased from Sigma-Aldrich (St. Louis, MO, USA) unless otherwise indicated.

### Plant material and growth conditions

The experiments were performed on etiolated wheat (*Triticum aestivum* L.) seedlings, which were grown hydroponically on Hoagland medium (0.16 g L^-1^; Hoagland’s No. 2 Basal Salt Mixture) in the dark for 6 days at 22±1°C. In some experiments seedlings were first deetiolated and then cut for experiments. Deetiolation was carried out under white light in one of the following conditions: (1) continuous light (100 μmol photons m^-2^ s^-1^) for a specific time period of 30 minutes to 24 hours; (2) a single flash from a photographic lamp (Quantuum MOVE 200, China; energy output 200 J) followed by incubatedion in the dark for 30 minutes or (3) a single flash from the photographic lamp followed by incubation in continous light (100 μmol photons m^-2^ s^-1^) for 30 minutes. For experiments, 2 cm-long-fragments from the tip, middle and basal parts of the leaves were cut and analyzed separately, as described in the Results. All manipulations connected with leaf harvesting and sample preparation were carried out in dim green light which did not cause any detectable amount of Chlide to be produced. Control (green) seedlings were grown under a 14-hour photoperiod (100 μmol photons m^-2^ s^-1^).

### Extraction of soluble proteins (crude extract) from leaf fragments

The basal (B), middle (M) and tip (T) fragments of the leaves were cut and used immediately for extracting soluble proteins according to the method described by [66].

The protein concentration was determined according to [97] using Bradford Reagent following the manufacturer's instructions. Bovine serum albumin was used as a standard. Absorbance at 595 nm was measured using a UV-Vis spectrophotometer Jasco V-650 (Jasco Co, Japan).

### Native PAGE, staining of SOD isoforms and SOD activity

Native polyacrylamide gel electrophoresis (PAGE) was carried out according to [66]. Protein extracts were mixed with glycerol (2:1 v/v) and stained with bromophenol blue before being applied to gel lanes. Extracts of equal protein content, within a range of 5–25 μg, were applied to each lane. Electrophoresis was carried out at 4°C and at 180 V for about 60 minutes.

SOD activity in the gels was detected using activity staining as described earlier [98]. The gels were incubated in a standard staining phosphate buffer (50 mM, pH 7.8), containing EDTA (1 mM), NBT (250 μM), TEMED (2.8 mM), riboflavin (22 μM) in darkness for 25 minutes. Then, they were exposed to artificial light using the LED panel illuminator (white SL 3500, 500–1000 μmol photons m^-2^ s^-1^ PSI, Czech Republic) until the SOD bands became visible. In the case of gels used to identify SOD isoforms, H_2_O_2_ (5 mM, final concentration) was added to the staining buffer to inhibit the Cu/Zn-SOD and Fe-SOD isoforms, whereas NaCN (2 mM) was added to inhibit solely Cu/Zn-SOD. Finally, the gels were washed three times with distilled water. The gels were scanned densitometrically using a TLC scanner Visualizer (Camag, Switzerland). Various forms of SOD activity were assessed using ImageJ (NIH, USA) and expressed in arbitrary units (a.u.) per 1 μg of protein.

### Plastid isolation

Plastids (etioplasts, etiochloroplasts or chloroplast) were isolated according to the method described earlier [99] with some modifications. The middle fragments (of about 3-cm in length, a total of 20 g) of leaves were cut into small pieces to 150 mL of a Hepes-NaOH buffer (25 mM, pH 7.5), containing 0.4 M sorbitol, 1 mM MgCl_2_, 1 mM EDTA and homogenized 3–5 times for around 20 seconds using a kitchen blender (easy power TYPE Y45 blender, Moulinex, France). The homogenate was gently filtered and centrifuged at 500 *g* and 4°C for 5 minutes. The supernatant was then centrifuged at 2100 *g* and 4°C for 10 minutes. The pellet was washed in isolation buffer and the centrifugation was repeated. The plastid-containing pellet was used for low temperature spectra and for SOD analysis. All manipulations were carried out under dim green light.

### Pigment extraction and quantification

The basal (B), middle (M) and tip (T) fragments of the leaves (10–15 fragments, in total ~ 0,1 g) were cut into small sections, ground to a powder in liquid nitrogen with MgCl_2_ and CaCO_3_, and immediately used for pigment extraction with 80% acetone. The pigment content was measured spectrophotometrically using a UV-Vis spectrophotometer Jasco V-650 (Jasco Co, Japan). Chls and carotenoids content was calculated according to the method that has already been described [100]. Pchlide content was calculated using the molar etinction coefficient determined by Kahn [101].

### Fluorescence spectroscopy

Fluorescence emission spectra were measured using a steady-state Perkin Elmer LS-55B spectrofluorimeter (Perkin Elmer, UK) equipped with a liquid nitrogen device for measurements at 77K. The excitation wavelength was 440 nm. The emission spectra were measured within a range 600–790 nm and automatically corrected for the wavelength-dependent sensitivity of the photomultiplier. The excitation and emission slits were set at 10 and 5 nm, respectively. A scanning speed of 500 or 700 nm/min was applied. Wavelength reproducibility was ± 0.5 nm.

The leaf fragments or etioplast suspension used for fluorescence measurements were placed in a glass capillary of 2.5 mm in diameter and 7 cm in length under dim green light and frozen in liquid nitrogen for 77 K fluorescence measurements.

### Statistical analysis

Approximately 150 seedlings grown under the same conditions were taken to prepare the pooled protein extract. All experiments were repeated 3–5 times. A one-way analysis of variance ANOVA and Tukey's post test were used for statistical analyses.

In the case of 77 K fluorescence experiments, B, M and T fragments of a single leaf were measured. The measurements were repeated for at least 16 leaves for etiolated plants and 11 leaves in the case of greening. See the description of the figures for more details. The statistical significance of these results was determined based on Student’s *t*-test with the Cochran-Cox adjustment when applicable. To determine if the Cochran-Cox adjustment is needed, Snedecor’s *F*-test of the equality of variances was used.

## Results

### Detection and identification of SOD forms

**Fig 1:**
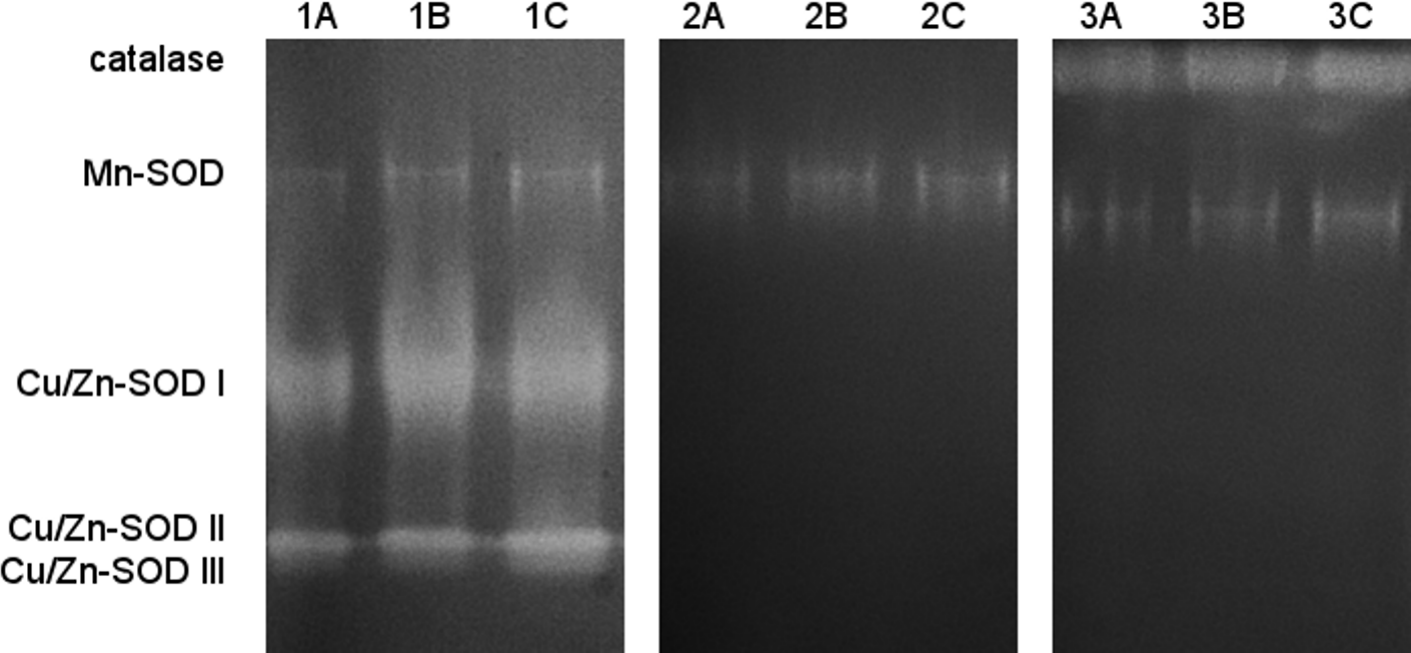
Identification of SOD forms in wheat leaves. Native polyacrylamide gel electrophoresis (PAGE) of SODs in 6-day-old wheat leaves: Lane 1A –etiolated, Lane 1B –etiolated and then deetiolated under white light (100 μmol photons m^-2^ s^-1^) for 4 hours, Lane 1C –grown under a 14 hour photoperiod (100 μmol photons m^-2^ s^-1^). The inhibitors were: NaCN–the inhibitor of Cu/Zn-SOD (Lanes 2A-2C) and H_2_O_2_ –the inhibitor of Fe-SOD and of Cu/Zn-SOD (Lanes 3A-3C). Each well was loaded with 25 μg of protein.

We analyzed protein extracts from whole leaves that were grown in different light conditions *i*.*e*. etiolated, greening and green leaves. Four SOD bands were observed in all these samples, as shown in Fig 1. The same pattern was found for different deetiolation times (S1 Fig). The specific reactions with NaCN, the inhibitor of Cu/Zn-SOD, and with H_2_O_2_, the inhibitor of Fe-SOD and of Cu/Zn-SOD, revealed the presence of two classes *i*.*e*. Mn-SOD and Cu/Zn-SOD. Among Cu/Zn-SOD, three isoforms were detected, denoted as I, II, III. Fe-SOD was not found (Fig 1). Cu/Zn-SOD I showed the highest activity in all the lanes, then Cu/Zn-SOD II form and the least Mn-SOD and Cu/Zn-SOD III.

### Distribution of SOD isoforms along a *T*. *aestivum* leaf

**Fig 2:**
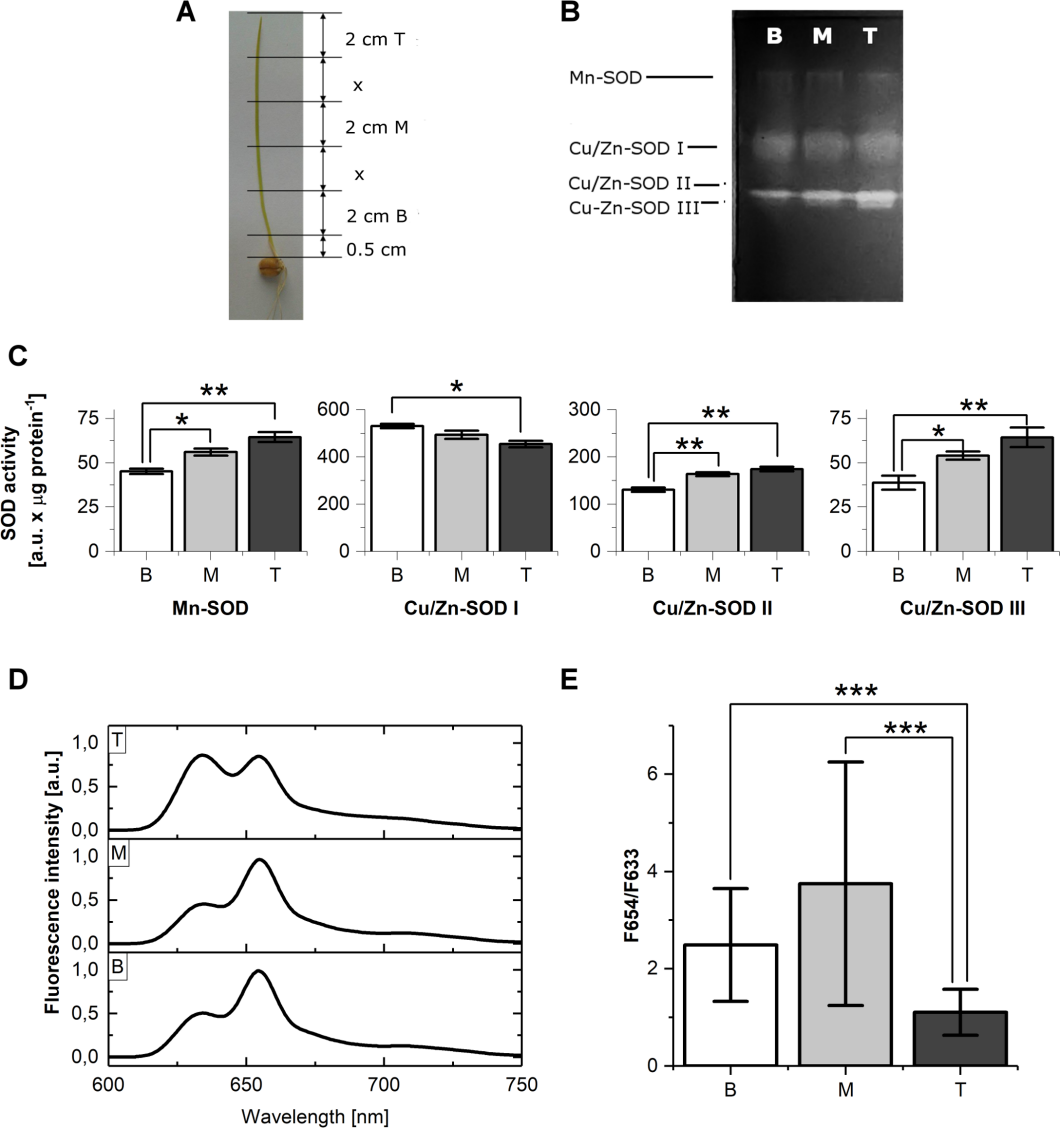
Distribution of SOD isoforms and Pchlide fluorescence along etiolated wheat leaves. A) Experimental model: tip (T), middle (M) and basal (B) leaf fragments were collected for the experiments; B) Native PAGE for the detection of SOD in T, M and B leaf fragments; the pooled sample was used for electrophoresis. Each well was loaded with 25 μg of protein; C) The distribution of the relative activity of SOD isoforms in B, M and T etiolated leaf fragments; the data represents the mean ± SD, n = 3, * p < 0.05, ** p < 0.01; D) 77 K fluorescence emission spectra of T, M and B etiolated leaf fragments; excitation: 440 nm; E) The average ratio of fluorescence intensity at 654 nm to the intensity at 633 nm (F654/F633) read from fluorescence spectra measured for single leaf fragments, the data represents mean ± SD, n > 30, *** p < 0.001.

The activity of SODs in the basal (B), middle (M) and tip (T) fragments of leaves (Fig 2A) was compared. Mn-SOD, Cu/Zn-SOD I, Cu/Zn-SOD II and Cu/Zn-SOD III isoforms were detected in all samples (Fig 2B). The highest activity was observed for Cu/Zn-SOD I, then for Cu/Zn-SOD II. The intensity of the corresponding bands differed among lanes representing different leaf fragments. An increase in the activity of Mn-SOD and of two Cu/Zn-SOD isoforms, *i*.*e*. Cu/Zn-SOD II and Cu/Zn-SOD III, from the base to the tip leaf fragments, was observed (Fig 2C). The activity of these enzymes was at least 30% higher in samples prepared from T fragments as compared to those prepared from B fragments. On the contrary, the activity of Cu/Zn-SOD I was at its highest in the B fragments and it decreased by about 20% toward the T fragments. The total activity of SODs was slightly higher for M leaf fragments than for T and B. However, this difference between samples always ranged between 2 and 10%.

Fluorescence emission spectra measured at 77 K revealed fluorescence properties of Pchlide accumulated in B, M and T fragments. They were complex (Fig 2D) and comprised two main bands having maxima at 633 and 654 nm, which originated from nonphotoactive and photoactive Pchlide, respectively. The relative fluorescence intensity of these bands expressed as a ratio of the intensity read from the spectra at 654 to that read at 633 nm (F654/F633) was at its highest in the M fragments, but at its lowest in the T fragments (Fig 2E). The high variability of this parameter among single leaves should be noted, which is due to a developmental heterogeneity among leaves and which points to similar heterogeneity in the pooled sample used for the analysis of SOD activity.

Measurements of pigment content revealed a gradient of carotenoids and Pchlide distribution along etiolated leaves (Table 1). For both pigments, the content was at its highest in T parts, while it was at its lowest in B parts. In the case of T fragments the high content of Pchlide (Table 1) and the highest fluorescence of the short-wavelength band (Fig 2D) corresponded to the highest activity of Mn-SOD, Cu/Zn-SOD II and Cu/Zn-SOD III found in these fragments (Fig 2C). However, there were no correlation between the activity of any SODs (Fig 2C) and the relative amount of photoactive Pchlide, expressed as F654/F633 ratio (Fig 2E) along a leaf.

**Table 1:** Protochlorophyllide and carotenoid content determined in T, M and B fragments of etiolated wheat leaves.

Leaf fragment	Pchlide [μg/g F.W.]	Carotenoids [μg/g F.W.]	Pchlide/Carotenoids
**T**	39.83±8.62	215.87±5.04	0.185±0.044
**M**	29.92±4.39	148.85±16.62	0.202±0.026
**B**	10.11±2.50	30.96±4.47	0.324±0.042

Statistical significance: for Pchlide content in T vs. B and M vs. B, p < 0.01; for Carotenoid content T vs. M, p < 0.05; T vs. B and M vs. B, p < 0.001, for Pchlide/Carotenoids T vs. B, p < 0.05; M vs. B p < 0.01; n = 3.

### Increase in SOD activity in flash-illuminated etiolated leaves

**Fig 3:**
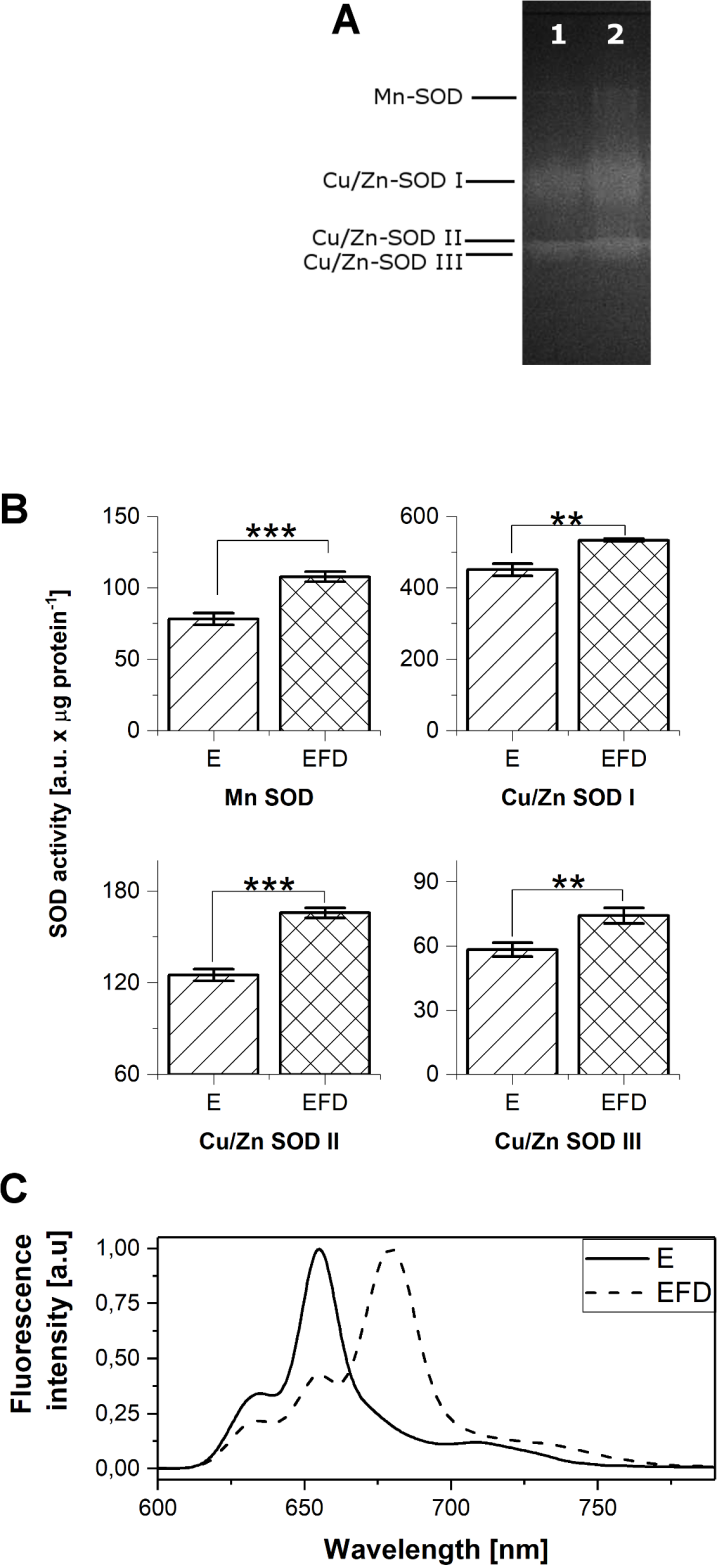
Flash-induced Pchlide photoreduction in 6-day-old etiolated wheat leaves. A) Native PAGE of SOD isoforms in M fragments of leaves: E—etiolated, EFD—etiolated and then illuminated with a single flash followed by incubation in darkness for 30 minutes; The pooled sample was used for electrophoresis; each well was loaded with 25 μg of protein B) The distribution of the relative activity of SOD isoforms in M leaf fragments; the data represents the mean ± SD, n = 3 (** p < 0.01; *** p < 0.001); C) Fluorescence emission spectra measured at 77 K for M fragments of laves; Excitation 440 nm.

In the case of plants that were illuminated with a strong flash of white light of one millisecond’s duration, and then incubated in the dark for 30 minutes, higher activity of all SOD forms was detected in M fragments as compared to the respective etiolated leaf fragments (Fig 3A and 3B). The increase of the activity of Cu/Zn-SODs was also noticed in T fragments in these experimental conditions, however, the effect was small. The respective low temperature fluorescence spectrum had the main band with a maximum at around 684 nm (Fig 3C). Two other bands at 633 nm and 654 nm were also seen, but their intensity was relatively low. In the case of spectra measured immediately after the flash, the disappearance of the emission band with a maximum at around 654 nm and the appearance of a new band at around 688 nm were observed (S2 Fig).

### Seedling deetiolation under continuous light

The greening process under continuous white light of 100 μmol photons m^-2^ s^-1^ was obviously the fastest in the M leaf fragments as compared to the B and T fragments. The M fragments became pale green-yellow after as little as 2 hours of greening whereas the T and B fragments stayed yellow and pale yellow, respectively. After 4–6 hours of continuous greening only the T fragments were still yellowish, whereas both B and M fragments were already green. After 24 hours, whole leaves became green.

**Fig 4:**
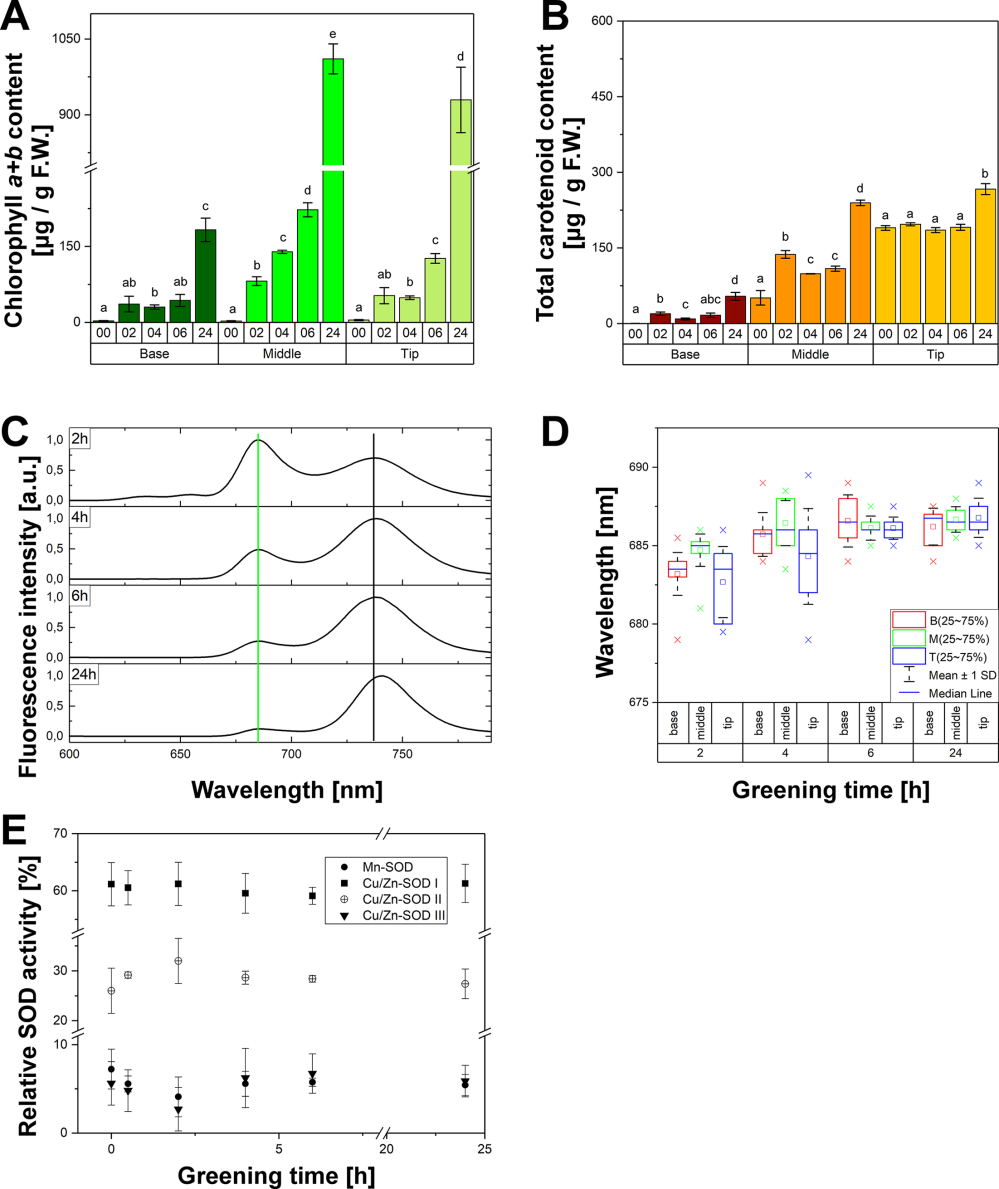
Deetiolation of 6-day-old etiolated wheat leaves under continuous white light (100 μmol photons m^-2^ s^-1^). A) Chlorophyll content; B) Carotenoid content; Statistically insignificant differences in A and B (p > 0.01) were marked with the same letters (n = 3); C) Representative 77 K fluorescence emission spectra for M leaf fragments; Excitation at 440 nm; D) Scatter of the position of fluorescence emission maximum (indicated with a green grid line in C) in the course of greening, as representative to the pooled sample taken for SOD native PAGE analysis; E) Average relative activity of SODs for M leaf fragments calculated with respect to the total SOD activity in a single lane of gel; 3–5 gels were compared for each greening time; the data represents the mean ± SD, n = 3–5.

These observations were confirmed by measuring photosynthetic pigment content. A massive accumulation of Chls was observed (Fig 4A). The amount of Chls in M fragments was always higher than in other fragments. The amounts of carotenoids increased over the course of greening in B and M parts (Fig 4B). In T parts, the amounts of carotenoids stayed approximately at the same level for up to six hours of greening and it was about 40% higher after 24 hours of greening. In all cases, the carotenoid content was at its highest in the T fragments and at its lowest in the B fragments.

The fluorescence emission spectrum measured at 77 K for M leaf fragments that had been deetiolated for different time periods under continuous light contained two bands, one with a maximum at around 680–685 nm and the other at around 730 nm (Fig 4C). Even if quite a large developmental heterogeneity was observed (Fig 4D), especially for T leaf fragments and short deetiolation times, the short-wavelength band showed a gradual red-shift for increasing greening times (Fig 4C and 4D). A similar effect was noticed for the other band (Fig 4C). The relative fluorescence intensity of the band with a maximum at around 730 nm significantly increased with increasing deetiolation time. A red-shift was also observed. This increase was much higher than that resulting only from self-absorption caused by the increasing amount of accumulated Chls (Figure A in S1 File).

Some fluctuations in the relative activity of SODs were observed in these greening conditions, especially for greening times shorter than 6 hours, and for M and T leaf fragments. For samples analysed on the same PAGE gel, a slight decrease in relative Mn-SOD activity was observed for an increasing deetiolation time. An increase in Cu/Zn-SODs activity (up to 20%) was also noticed at the beginning of greening with respect to the etiolated samples. However, because of the limited number of lanes on a single gel it was impossible to compare samples directly for all these greening times. To compare all the results, we normalized the activity of all the isoforms in a single lane and compared the relative activities of the isoforms (Fig 4E**)**. The decrease in the relative activity of Mn-SOD and Cu/Zn-SOD III, and the increase in this activity for Cu/Zn-SOD II were noticed within the first 2 hours of deetiolation.

It has to be noted that in the case of samples grown under photoperiod, the activity of Mn-SOD was significantly higher (about 80%, p < 0.05) whereas the activity of Cu/Zn-SOD II was significantly lower (30%, p < 0.05) as compared to samples that were etiolated and then deetiolated for 24 hours.

### The activity of SOD in isolated plastids

**Fig 5:**
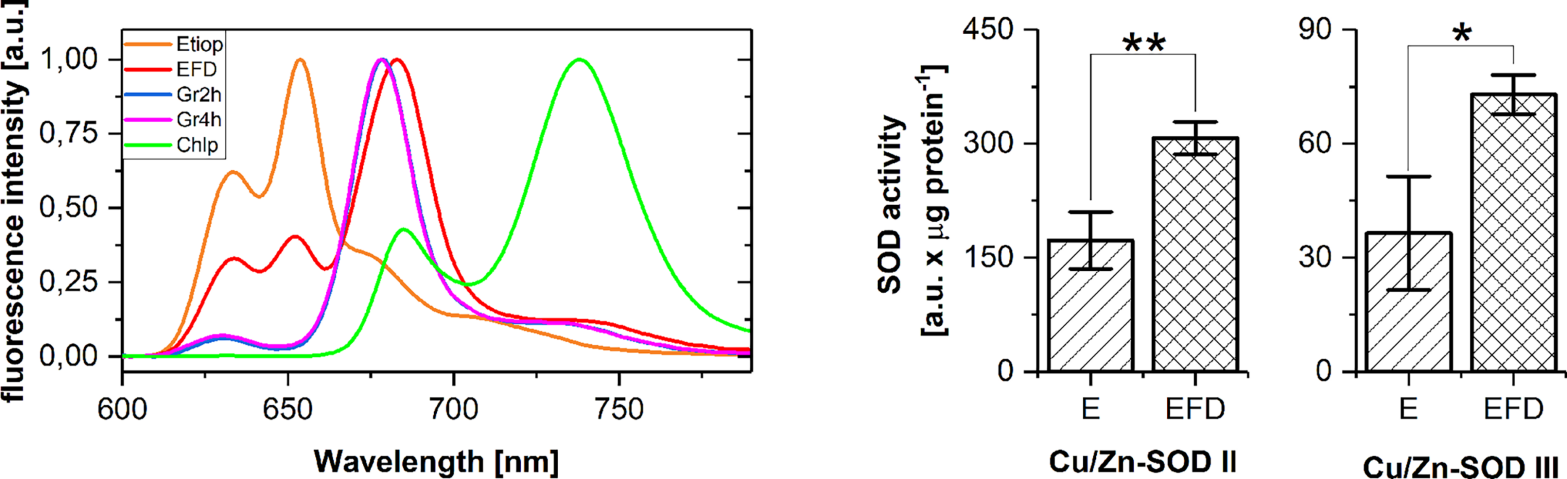
SOD in isolated plastids. A) Fluorescence emission spectra measured at 77 K for plastids isolated from leaves treated in the same way as for SOD identification; excitation: 440 nm; greening time–indicated in the legend; EFD–plastids isolated from leaves that were etiolated for 6 days and then illuminated with a flash of white light; plastid isolation was begun directly after the flash or indicated greening times and lasted c.a. 30 min; they were isolated under a dim green light; B) The distribution of the relative activity of SOD isoforms in plastids isolated from M leaf fragments; the data represents the mean ± SD (n = 3, * p < 0.05; ** p < 0.01).

To monitor the abundance of SODs in developing chloroplasts, etioplasts were isolated from etiolated seedlings, and etiochloroplasts were isolated from seedlings that had been etiolated and then flashed and incubated in the dark, as well as from those that had been etiolated and deetiolated under continuous light. Greening conditions were the same as those used in the study of leaves, as described above. Aliquots of the samples taken for SOD analysis were used for recording fluorescence emission spectra at 77 K. Three fluorescence bands were observed in the spectrum measured for etiochloroplasts that were isolated from leaves after illumination with a single flash followed by dark incubation (Fig 5A). The band at 655 nm was not observed for deetiolation under continuous illumination, thus it originated from regenerated photoactive Pchlide complexes. The main band observed for etiochloroplasts at 680–682 nm was accompanied by two bands of low intensity with maxima at 633 and 730 nm. In the case of chloroplasts, the fluorescence band with a maximum at 730 nm showed much higher intensity than the band at 680 nm.

In isolated plastids, only Cu/Zn-SOD II and Cu/Zn-SOD III isoforms were detected (Fig 5B). The highest activity of these enzymes was noticed for the samples that were illuminated with a flash and then kept in darkness. For plastids isolated from leaves that were deetiolated under continuous light, no significant differences in SOD activity were observed.

## Discussion

Our study showed the presence of the same SOD forms, *i*.*e*. one Mn-SOD and three isoforms of Cu/Zn-SOD, in etiolated, green and greening leaves of *T*. *aestivum* (Fig 1, S1 Fig). This means that no additional SOD forms appear due to a light-triggered change of plant developmental program from skoto- to photomorphogenesis. Cu/Zn-SOD II and Cu/Zn-SOD III were found in plastids (Fig 5), which is in line with the earlier studies [60–62]. Cu/Zn-SOD II showed much higher activity than Cu/Zn-SOD III. Fe-SOD, which is also regarded as a chloroplastic enzyme [62,71], was not detected in wheat samples in this study. Similarly, Navari-Izzo et al. [60] detected only Cu/Zn-SODs in washed wheat chloroplasts. Recently, a weak expression of *FeSOD* was shown in etiolated wheat seedlings, which strongly increased during deetiolation [102]. However, the activity of Fe-SOD stayed undetectable on the native gels up to 48 hours of deetiolation. It was then concluded that Fe-SOD isoform is characteristic for mature chloroplasts [102].

We found Cu/Zn-SOD I only in samples prepared from leaves (Figs 1–3) but not in isolated plastids (Fig 5). As has already been shown [62], it seems to be an extraplastidic SOD form, probably a cytosolic one. The observation of Mn-SOD in leaves (Figs 1–3) and not in the isolated plastids (Fig 5**)** is in agreement with the already confirmed mitochondrial and peroxisomal localization of this enzyme [47,73].

The activity of different SOD forms was unequally distributed along etiolated wheat leaves (Fig 2B and 2C). Obviously in monocot plants, B leaf fragments contain the youngest, dividing cells, whereas the oldest cells are in T fragments [22,95,96]. The increase in Mn-SOD activity observed from the base to the tip of leaves indicates that ROS production increases in peroxisomes or in the respiratory chain in mitochondria in aging *T*.*aestivum* mesophyll cells. The increase in the activity of plastidic Cu/Zn-SOD II and Cu/Zn-SOD III from the base to the tip of leaves fragments, pointed to an increasing ROS production in aging etioplasts, which accumulate a high amount of Pchlide, much higher than those in B parts (Table 1). Although, the amount of Pchlide accumulated in M fragments was only about 25% lower than in T fragments, the relative amount of photoactive Pchlide was much higher there, as judged from F654/F633 parameter (Fig 2D and 2E). This reflects the readiness of M fragments to become green upon illumination. Moreover, the accumulation of photoactive Pchlide:LPOR:NADPH complexes is an indirect confirmation of the presence of a regular PLB structure in M leaf fragments [16,95,103,104]. On the contrary, T fragments accumulated mostly nonphotoactive Pchlide (Table 1 and Fig 2E). This may be related to insufficient LPOR delivery or partial degradation in the photoactive Pchlide:LPOR:NADPH complexes and PLBs in aging etioplasts. A decrease of the F654/F633 ratio and loss of the ability to convert Pchlide into Chlide was already induced by thermal deactivation of the LPOR enzyme in isolated PLBs [105] as well as by NADPH oxidation and PLB degradation caused by Hg^2+^ [106,107] and other ions [108]. To sum it up, the observation of an increase in the activity of plastidic Cu/Zn-SOD II and Cu/Zn-SOD III from the base to the tip of leaves would point to a source of light-independent ROS formation in aging etioplasts. This is somehow connected to the accumulation of nonphotoactive Pchlide and does not correspond to the ability to green in respective leaf fragments, as reflected by the F654/F633 ratio.

Interestingly, we observed a significant decrease in Cu/Zn-SOD I activity, which is an extraplastidic SOD form, toward the tip of etiolated leaves (Fig 2C), which was even higher for green leaves (S3 Fig). This observation indicates high ROS production in the cytosol or apoplast of young cells, which may be linked to biochemical processes related to cell differentiation in monocot leaves.

The next question we addressed in this study concerned SOD activity at initial deetiolation. At this developmental stage, juvenile plants are illuminated unexpectedly for the first time, which may induce unquenched overexcitation of tetrapyrroles, in particular Pchlide and Chlide, and brings the risk of ROS formation. A significant increase in the activity of all SOD forms was observed shortly after the pulse of light followed by dark incubation, indicating an increase in ROS production. The effect was noticed mainly in M leaf fragments (Fig 3B) and in isolated etioplasts (Fig 5B). The flash triggered the photoreduction of photoactive Pchlide. In the following dark incubation, the nonphotoacive Pchlide served as a substrate for the regeneration of photoactive Pchlide:LPOR:NADPH complexes manifested in our experiments as a band around 654 nm in the illuminated samples (Figs 3C and 5A), which concurs with earlier observations [20,21]. Moreover, judging from the position of Chlide fluorescence maximum (Figs 3C and 5A) [21], we can state that Chlide release was already well-advanced at the end of dark incubation, and thus in the samples taken for SOD analysis. According to the earlier observations [17,21], this Chlide probably underwent esterification to Chl in our samples. However, it should be mentioned that the flash also excited molecules of nonphotoactive Pchlide present in the samples (Figs 2D, 2E and 5A). They may interact with oxygen, leading to singlet oxygen formation and inducing photooxidative damage to the etiolated seedlings [79,109,110]. On the other hand, it was shown that singlet oxygen does not act primarily as a toxin but rather as a signaling molecule that activates several stress-response pathways, different from those induced by superoxide or H_2_O_2_ [79]. Our experiments clearly showed the highest increase in SOD activity in samples where the excitation light was efficiently used for Pchlide photoreducion to Chlide and the relative content of nonphotoactive Pchlide was quite small. This indicates the involvement of SODs in ROS scavenging in flash-illuminated and subsequently dark-incubated etioplasts, probably to protect the newly formed Chlide.

It should also be underlined that inducing deetiolation with a single flash followed by dark incubation is a model frequently used for studying the initial reactions of the deetiolation process [10,11,22]. However, care should be taken concerning ROS formations, which may be particularly important for plant species that accumulate a relatively high level of nonhotoactive Pchlide form.

SOD activity was also monitored in wheat seedlings over the course of their deetiolation under continuous white light of moderate intensity (100 μmol photons m^-2^ s^-1^). It is well known that continuous illumination results in a continuous Pchlide photoreduction and Chl formation, which triggers PLB dispersion followed by formation of thylakoid membranes. The greening process is multiphasic and the individual mechanisms involved in energy trapping, protection as well as respiration are sequentially activated [21,99,111–114]. First, the photosystems reaction centre cores are assembled, which is followed by a progressive development of complex antenna systems. It was shown that the assembly of the PSII activity started within the first 20 minutes of greening [83] and took several hours to be completed [83,111]. The formation of PSII reaction centers and their minor antenna components were observed within the first 3–4 hours of deetiolation, whereas LHCII polypeptides started to accumulate after that time [99]. However, at the early greening stages an unbound Chl was detected, which could be detected for up to 7 hours of greening [83]. Chl molecules unbound to pigment-protein complexes showed photophysical properties similar to Chl monomers in solution [83], and can easily undergo photooxidation [115].

In our study, spectral changes reflecting the assembly of PS I and PS II complexes were observed mainly within the first four hours of greening (Fig 4C and 4D**).** For these greening stages, some fluctuations in the relative activity of plastidic Cu/Zn-SOD II and Cu/Zn-SOD III were observed (Fig 4E). For deetiolation longer than 4 hours, a gradual increase in the relative fluorescence intensity of the band with its maximum around 730 nm (Fig 4C**)**, which reflected the ongoing assembly of the PSI complexes. However, the relative activity of SODs stayed at the same level for greening times longer than 4 hours (Fig 5E).

Garmash et al. [102] showed a gradual increase of total SODs activity over the course of 12 hours of wheat greening, although the activity of Cu/Zn-SOD II isoform stayed at the same level. The activity of Cu/Zn-SOD I was not detected after 24 hours of greening. Interestingly, the relative expression of *CuZnSOD* was significantly lower in leaves after one hour of deetiolation as compared to etiolated ones, and gradually increased up to 12 hours of deetiolation. Mattagajasingh and Kar [116] observed unchanged total SOD activity during the greening of etiolated wheat leaves. However, it is difficult to compare directly the greening condition in those studies and in our own. An increase in total SOD activity was observed over the course of the deetiolation of oat and pea plumules, being at its highest within the first 12 hours, although they did not analyse greening times shorter than 12 hours [117]. Moreover, an increase in activity of other antioxidant enzymes (catalase, peroxidase and glutation reductase) was also observed during greening of wheat seedlings [102,116].

ROS production at the initial greening stages under continuous light may also be related to a developing and not fully functioning electron transport chain in etiochloroplasts. The developing PSI may be a site of O_2_^•-^ generation similar to green plants [71,118]. In the case of overloading the electron transport chain and NADP^+^ deficiency, some electrons are transported from ferredoxin to O_2_, reducing it to O_2_^•-^via a Mehler reaction [119]. The acceptor site of PSII can also participate in the leakage of electrons to molecular oxygen, resulting in the formation of a O_2_^•-^ [120–123]. It was recently shown that plastid terminal oxidase, which may play a role in the reoxidation of reduced PQ [124–126], is essential for chloroplast biogenesis [127], especially during the early stages of chloroplast biogenesis prior to the development of full photosynthetic competence [128]. However, it can also act as a site of O_2_^•-^ production in a side reaction [129]. Plastidic SODs forms play a role in scavenging O_2_^•-^ produced in these reactions. Thus we observed changes in SOD activity at initial greening. However, the specificity of each of two plastidic SODs is still unknown.

This study has shown the involvement of SODs in the process of angiosperm greening. Greening under continuous light of moderate intensity may induce ROS generation at the early greening stages, covering the assembly of the photosystems and minor antenna, as shown by changes in the activity of SODs. In contrast to this, a single flash induced a significant increase in SOD activity even if the Pchlide photoreduction was efficient. It should be underlined that triggering the deetiolation with a single flash and with a continuous illumination might differently influence the signalling pathways controlled by phytochromes and other photoreceptors. The results of this study also showed that triggering deetiolation with a strong flash, which is often used in greening studies, needs to be revised with respect to uncontrolled ROS generation.

## Supporting information

S1 Fig SOD forms in wheat leaves. Native PAGE of SODs in 6-day-old wheat leaves: Lanes 1, 2, 3 M, T, B leaf fragments; A- etiolated; B, C, D–etiolated and then deetiolated under white light (100 μmol photons m^-2^ s^-1^) for 4 hours (B), 6 hours (C), 24 hours (D). Each well was loaded with 25 μg of protein.
(TIF)

S2 Fig Fluorescence spectra of etiolated leaf after a flash. A representative 77K fluorescence spectrum of etiolated M fragment treated with a single flash of white light from a photographic lamp (Quantuum MOVE 200, China; energy output 200 J)) and immediately frozen at liquid nitrogen. The bands of nonphotoactive Pchlide and the newly formed Chlide are indicated.
(TIF)

S3 Fig Cu/Zn SOD I activity in green wheat leaves. The distribution of the relative activity of Cu/Zn-SOD I isoform in B, M and T leaf fragments of seedlings grown under a 14-hour photoperiod (100 μmol photons m^-2^ s^-1^); The data represents the mean ± SD, n = 3, ** p<0.01.
(TIF)

S1 File Self-absorption problem in 77 K fluorescence measurements. (DOCX)

## Abbreviations

Chlide: chlorophyllide
Chl: chlorophyll
F.W.: fresh weight
LPOR: light-dependent protochlorophyllide oxidoreductase
Pchlide: protochlorophyllide
PLB: prolamellar body
ROS: reactive oxygen species
SOD: superoxide dismutase
