# Supplementary material for: The activity of superoxide dismutases (SODs) at the early stages of wheat deetiolation
Source: PLoS One. 2018 Mar 20;13(3):e0194678. doi: 10.1371/journal.pone.0194678 (PMC5860746; doi:10.1371/journal.pone.0194678)
Supplement: S1 File — (DOCX) [file pone.0194678.s004.docx]

**Self-absorption problem in 77 K fluorescence measurements**

The highest increase in Chl content was noted in the deetiolation of M leaf fragments (Fig. 4A). The Chl content increased around 100 times during the 24-h greening period. At the same time, the 77 K fluorescence spectra showed an increase in the relative intensity of the band with its maximum around 730 nm, which originates from PSI fluorescence (Fig. 4C). However, bearing in mind that Chl content increased among samples measured for increasing greening times, the fluorescence signal might be distorted by the reabsorption of the emitted light.

To estimate the influence of Chl self-absorption on the fluorescence intensity measured at 77 K for greening leaves, we carried out additional experiments. Fragments of green wheat leaves were ground to a powder in liquid nitrogen and resuspended with precooled buffer: glycerol (80:20 v/v) mixture. The buffer was 25 mM Hepes-NaOH, pH 7,5 containing 0,4 M sorbitol, 1 mM MgCl2 and 1 mM EDTA. 200ul of this suspension was used for extracting pigment with 80% acetone. Then, the Chl content was measured spectrophotometrically using a Jasco V-650 UV-Vis spectrophotometer and calculated as described in Material and Methods. The rest of the suspension was used to prepare a series of dilutions to obtain samples with a Chl concentration corresponding to those determined for the M leaf fragments during their deetiolation. Then, fluorescence emission spectra were measured for the series of dilutions using a steady-state Perkin Elmer LS-55B spectrofluorimeter (UK) equipped with a liquid nitrogen device for measurements at 77K. The excitation wavelength was 440 nm. The emission spectra were measured within a range 600 - 790 nm and automatically corrected for the wavelength-dependent sensitivity of the photomultiplier. The excitation and emission slits were set at 10 and 5 nm, respectively and the scanning speed was 500 nm/min.

The fluorescence intensity at 730 and 685 nm was read from the spectra and the fluorescence ratio of F730/F685 nm was calculated. The dependence of F730/F685 vs Chl concentration is shown below:

**Figure A) F735/F685 ratio calculated for fluorescence emission spectra measured for a series of dilutions of leaf homogenate, as described in the text.**

Of the different greening times, the highest distortion caused by self-absorption is expected for 77K fluorescence spectra measured for M leaf fragments that were deetiolated for 24 h. In this case the F730/F685 ratio calculated for the measured spectra was 7±1. The estimated maximal distortion originating from self-absorption is around 2. Therefore, in the light of the above considerations, our interpretation regarding PSI development over the course of deetiolation is valid.
